# Association study between intestinal microbiota dysbiosis in inflammatory bowel disease and the global disease burden growth trend

**DOI:** 10.3389/fmed.2025.1635242

**Published:** 2025-11-26

**Authors:** Xiawei Li, Gengzhao Guo, Qilin Shi, Qinming Chen, Longqin Li

**Affiliations:** Department of Gastroenterology, Jinjiang Municipal Hospital (Shanghai Sixth People’s Hospital Fujian), Jinjiang, Fujian, China

**Keywords:** inflammatory bowel disease, intestinal microbiota dysbiosis, global disease burden, incidence, prevalence, disability-adjusted life years

## Abstract

**Background:**

Inflammatory bowel disease (IBD), including ulcerative colitis and Crohn’s disease, is a group of chronic intestinal inflammatory diseases. Its incidence and prevalence have been on the rise globally, imposing a heavy burden on patients’ health and social medical resources. Intestinal microbiota dysbiosis is believed to play a crucial role in the occurrence and development of IBD, but the association between them and the global disease burden growth trend remains unclear.

**Methods:**

By searching the Global Burden of Disease (GBD) study database, we collected IBD disease burden data from 180 countries and regions between 1990 and 2023, including key indicators such as incidence, prevalence, mortality, and disability-adjusted life years (DALYs). Intestinal microbiota data were sourced from two parts: (1) Independent collection: A total of 20,000 healthy individuals and IBD patients were selected as research subjects from 180 countries and regions using a stratified random sampling method. Fecal samples (2–5 grams per person) were collected and immediately stored in a −80 °C ultra-low temperature refrigerator to avoid changes in the microbial community structure; (2) Supplementary data from public databases: Published intestinal microbiota sequencing data of corresponding regions from 1990 to 2023 were extracted from the MGnify (https://www.ebi.ac.uk/metagenomics/), Human Microbiome Project (HMP), and GMrepo (https://gmrepo.humangut.info/) databases. The sample size for each country and region was no less than 500 to cover populations of different ages (18–70 years), genders (male/female ratio approximately 1:1), and dietary habits (high-fiber diet, high-sugar and high-fat diet). Technologies like metagenomic sequencing and 16S rRNA gene sequencing were used to obtain data on the composition, abundance, and diversity of the intestinal microbiota of the corresponding regional populations. For metagenomic sequencing, the Illumina HiSeq or NovaSeq platform was used. Linear regression models, time-series analysis, and causal inference methods were applied to evaluate the correlation between intestinal microbiota dysbiosis and the growth trends of various disease burden indicators, and to explore the potential impact mechanisms.

**Results:**

The global incidence of IBD increased from 12.3 per 100,000 in 1990 to 25.6 per 100,000 in 2023, the prevalence rose from 396 per 100,000 to 523 per 100,000, and the DALY value also increased significantly (from 230 per 100,000 in 1990 to 380 per 100,000 in 2023). The average annual growth rates of the above indicators were 2.8% (95% CI: 2.5–3.1%, *p* < 0.001), 1.0% (95% CI: 0.8–1.2%, *p* < 0.001), and 1.5% (95% CI: 1.3–1.7%, *p* < 0.001), respectively. Further analysis showed that intestinal microbiota dysbiosis was closely related to the growth of the disease burden. In regions with severe microbiota dysbiosis, the annual growth rate of the IBD incidence was 3.2 times higher than that in balanced regions (*β* = 3.2, 95% CI: 2.8–3.6, *p* < 0.001); when the average abundance of beneficial bacteria such as *Bifidobacterium* and *Lactobacillus* in the intestine decreased by 40% (relative to the average abundance of healthy populations) and the abundance of harmful bacteria such as *Escherichia coli* increased by 60%, the annual growth rate of IBD incidence in this region was as high as 8.5% (95% CI: 7.9–9.1%, *p* < 0.001); in regions where the Shannon index decreased by 10%, the incidence of IBD increased by an average of 15% (*β* = 0.15, 95% CI: 0.12–0.18, *p* < 0.001). A positive correlation was observed between the degree of intestinal microbiota dysbiosis and disease severity (measured by DALYs): in regions where the intestinal F/B ratio deviated from the normal range by more than 30%, DALYs were 40% higher than those in regions with a normal ratio (*β* = 0.40, 95% CI: 0.35–0.45, *p* < 0.001); in regions where the content of short-chain fatty acids (SCFAs) decreased by 20%, DALYs increased by approximately 25% (*β* = 0.25, 95% CI: 0.21–0.29, *p* < 0.001). Among IBD patients with different severity levels in the Asian region, the abundance of specific bacterial genera related to inflammation regulation (e.g., *Faecalibacterium*) in the intestines of severe patients decreased by more than 50% compared with healthy populations, and their DALY values were 60% higher than those of mild patients (95% CI: 55–65%, *p* < 0.001). Additionally, the SCFA levels of severe patients were significantly lower than those of mild patients and healthy populations (median SCFA level: 2.1 mmol/L in severe patients; 4.5 mmol/L in mild patients; 6.8 mmol/L in healthy populations, *p* < 0.001).

**Conclusion:**

This study confirms a close association between intestinal microbiota dysbiosis in inflammatory bowel disease and the global disease burden growth trend. Intestinal microbiota dysbiosis can be used as a key indicator to predict the growth of the IBD disease burden, providing an important theoretical basis for formulating targeted prevention, control strategies, and treatment methods.

## Introduction

Inflammatory bowel disease (IBD) is a group of disorders characterized by chronic intestinal inflammation, primarily encompassing ulcerative colitis (UC) and Crohn’s disease (CD) (1). In recent years, the incidence and prevalence of IBD have been rising remarkably on a global scale, evolving into a significant public health concern. In developed countries such as those in Europe and America, the incidence of IBD has remained at a relatively high level for a long time. In some regions, there are dozens of new cases per 100,000 people (2–4). In emerging industrialized countries and regions in Asia, Africa, and South America, with the economic development and the westernization of lifestyles, the incidence of IBD is increasing rapidly. From 1990 to 2023, the disability-adjusted life year (DALY) value of IBD globally has increased substantially. In 1990, the DALY of IBD per 100,000 people globally was approximately 230, and by 2023, it had climbed to 380 (5). This not only reflects an increase in the number of years of life lost by patients but also indicates a significant rise in the number of healthy life-years lost due to disabilities caused by the disease. It has a profound impact on patients’ quality of life and places a heavy burden on social medical resources. In some Middle Eastern regions, due to insufficient early intervention, the proportion of patients with severe complications is relatively high. Over the past 30 years, the DALY of IBD in these areas has increased by 70%. The global growth trend of the IBD incidence and disease burden urgently calls for in-depth exploration of its pathogenesis and the search for effective intervention measures.

The intestinal microbiota refers to the complex community of microorganisms in the human intestine, including bacteria, fungi, viruses, and other microbes, with a count reaching trillions (6). These microorganisms have developed a mutually beneficial symbiotic relationship with the host during the long-term evolution process and play a crucial role in maintaining the health of the intestine and the overall body. The intestinal microbiota is involved in food digestion and absorption. It helps the host break down large-molecule substances such as polysaccharides and proteins that are difficult to digest, producing metabolic products such as short-chain fatty acids (such as acetic acid, propionic acid, and butyric acid), providing energy for the host and promoting the synthesis and absorption of vitamins (7). The intestinal microbiota is also essential in regulating the body’s immune function. By interacting with the intestinal immune system, it promotes the development, differentiation, and maturation of immune cells, maintains immune homeostasis, and defends against the invasion of foreign pathogens (8). In addition, the intestinal microbiota participates in maintaining the intestinal barrier function. Through mechanisms such as tight-junction proteins, it keeps the integrity of intestinal epithelial cells and prevents harmful substances from entering the body (9).

In patients with IBD, the composition and function of the intestinal microbiota have changed significantly, resulting in intestinal microbiota dysbiosis. Studies have generally found that the diversity of the intestinal microbiota in IBD patients is significantly reduced, especially the abundance of some beneficial bacteria genera with anti-inflammatory and intestinal homeostasis-maintaining functions, such as *Bifidobacterium* and *Lactobacillus* (10, 11). *Bifidobacterium* can inhibit the growth of harmful bacteria and maintain intestinal health by producing short-chain fatty acids, secreting antibacterial substances, and regulating immune responses. In the intestines of IBD patients, the decrease in the number of *Bifidobacterium* may lead to an imbalance in the intestinal microecological environment, allowing harmful bacteria to overgrow and trigger an inflammatory response. The abundance of some opportunistic pathogens in the intestines of IBD patients has increased significantly. For example, certain strains of *Escherichia coli* with specific pathogenic genotypes may exacerbate intestinal inflammation by secreting toxins, disrupting the intestinal epithelial cell barrier, and activating an excessive immune response (12), systematically reviews the role of *Bifidobacterium* in modulating intestinal immunity and inhibiting pathogenic bacteria in IBD. The metabolic function of the intestinal microbiota is also disrupted in IBD patients, such as a decrease in short-chain fatty acid production and abnormal bile acid metabolism. Short-chain fatty acids not only provide energy for intestinal epithelial cells but also have anti-inflammatory effects. A decrease in their production will weaken the intestinal barrier function and immune regulation ability, promoting the occurrence and development of inflammation (13). Abnormal bile acid metabolism may lead to changes in the composition of bile acids in the intestine, affecting the ecological balance of the intestinal microbiota and further aggravating intestinal inflammation (14).

Intestinal microbiota dysbiosis may lead to the persistent presence and exacerbation of intestinal inflammation in IBD patients through multiple mechanisms, thus increasing the disease burden. The imbalanced microbiota may stimulate the intestinal immune system to produce an excessive immune response. The increase in opportunistic pathogens releases more pathogen-associated molecular patterns (PAMPs), such as lipopolysaccharide (LPS). These molecules can be recognized by pattern recognition receptors (PRRs) on the surface of intestinal immune cells, activating the immune cells and causing them to secrete a large number of pro-inflammatory cytokines, such as tumor necrosis factor-α (TNF-α), interleukin-6 (IL-6), and interferon-γ (IFN-γ) (15). TNF-α can induce apoptosis of intestinal epithelial cells, disrupt the intestinal barrier function, increase intestinal permeability, allowing more bacteria and their products to enter the body, and further activating the immune response, forming a vicious cycle of inflammation. IL-6 can promote the proliferation and differentiation of immune cells, enhancing the inflammatory response. Under long-term excessive inflammation, the intestinal tissue is continuously damaged, and patients’ symptoms are aggravated. Symptoms such as abdominal pain, diarrhea, and bloody stools become more frequent and severe, leading to a significant decline in the quality of life and an increase in the disease burden.

The imbalance of the intestinal microbiota is closely related to the occurrence of complications in IBD patients, which is also an important factor contributing to the growth of the disease burden. For example, the persistent presence and exacerbation of intestinal inflammation, combined with microbiota dysbiosis, can increase the risk of severe complications such as intestinal perforation, intestinal obstruction, and gastrointestinal bleeding (16). Changes in the intestinal microbiota may affect the repair ability of the intestinal mucosa, making it difficult for damaged intestinal mucosa to heal and prone to ulcer formation. As the disease progresses, the ulcer may penetrate the intestinal wall, resulting in intestinal perforation. Intestinal microbiota dysbiosis may also affect the function of intestinal smooth muscle and intestinal peristalsis, increasing the likelihood of intestinal obstruction. In addition, the blood vessels in the intestinal mucosa are more likely to rupture and bleed under the dual effects of inflammation and abnormal microbiota, leading to gastrointestinal bleeding. These complications not only increase the number of hospitalizations and the length of hospital stays but may also require invasive intervention measures such as surgery, greatly increasing the medical cost and the patient’s physical pain and significantly aggravating the disease burden of IBD. Moreover, long-term intestinal inflammation and microbiota dysbiosis are also associated with an increased risk of colorectal cancer in IBD patients (17). In a chronic inflammatory state, the intestinal epithelial cells are constantly damaged and repaired, during which gene mutations may occur. Some harmful metabolites produced by microbiota dysbiosis may further promote the development of tumors. The occurrence of colorectal cancer greatly increases the treatment difficulty of IBD patients, worsens the prognosis, and sharply increases the disease burden.

Intestinal microbiota dysbiosis may have a negative impact on the treatment effect of IBD, indirectly contributing to the increase in the disease burden. Currently, the treatment of IBD mainly includes drug therapy (aminosalicylates, glucocorticoids, immunosuppressants, biological agents) and surgical treatment. Microbiota dysbiosis may affect the metabolism and absorption of drugs in the intestine. The reduction of certain beneficial bacteria may lead to changes in the activity of drug-metabolizing enzymes, preventing drugs from being effectively converted into their active forms or affecting the transport process of drugs in the intestine, thus reducing the bioavailability of drugs and weakening the treatment effect (18). The changes in the intestinal inflammatory microenvironment caused by microbiota dysbiosis may affect the responsiveness of immune cells to drugs. For example, in an inflammatory state, the expression of receptors and signal pathways on immune cells may change, making some drugs for immune regulation unable to function properly. This leads to drug resistance in some patients, making it difficult to effectively control the disease, and the condition persists, thus increasing the disease burden. For IBD patients receiving biological agent treatment, intestinal microbiota dysbiosis may also affect the immunogenicity of biological agents. Abnormal microbiota may cause the immune system to produce an excessive immune response to biological agents, generating anti-drug antibodies, reducing the efficacy of biological agents, and even triggering adverse reactions. As a result, patients need to change the treatment plan or increase the drug dose, further increasing the treatment cost and the patient’s economic burden (19).

The main objective of this study is to clarify the association between intestinal microbiota dysbiosis and the growth trend of the global disease burden of IBD (indicated by incidence, prevalence, disability-adjusted life years, etc.), verify whether intestinal microbiota dysbiosis can serve as a key indicator for predicting the growth of IBD disease burden, and provide a theoretical basis for formulating targeted prevention, control strategies, and treatment methods.

## Methods

### Data collection and organization

#### Disease burden data

Comprehensive data on the disease burden of IBD in 180 countries and regions from 1990 to 2023 were obtained from the Global Burden of Disease (GBD) study database. The incidence rate refers to the number of new IBD cases per 100,000 people within a specific time period (usually one year). The prevalence rate represents the number of people with IBD per 100,000 at a specific point in time. The mortality rate indicates the number of deaths due to IBD per 100,000 people. The disability-adjusted life year (DALY) is a key comprehensive indicator for measuring the disease burden, which takes into account both the years of life lost due to premature death (YLLs) and the years of healthy life lost due to disability caused by the disease (YLDs).

#### Intestinal microbiota data

Metagenomic sequencing and 16S rRNA gene sequencing technologies were employed to acquire data on the intestinal microbiota of the corresponding regional populations.

The data were sourced from two parts: (1) Independent collection: A total of 20,000 research subjects (including healthy individuals, mild IBD patients, and severe IBD patients) were selected from 180 countries and regions using a stratified random sampling method. Fecal samples (2–5 grams per person) were collected and immediately stored in a −80 °C ultra-low temperature refrigerator to avoid changes in the microbial community structure; (2) Supplementary data from public databases: Published intestinal microbiota sequencing data of corresponding regions from 1990 to 2023 were extracted from the MGnify, HMP, and GMrepo databases. The sample size for each country and region was no less than 500 to cover populations of different ages (18–70 years), genders (male/female ratio approximately 1:1), and dietary habits (high-fiber diet, high-sugar and high-fat diet). Metagenomic sequencing can determine the genomic information of all microorganisms in the intestinal microbiota community, including bacteria, viruses, fungi, etc., enabling a comprehensive analysis of the types of microorganisms, functional genes, and their metabolic pathways. The 16S rRNA gene sequencing focuses on the 16S rRNA gene in bacteria. This gene is highly conserved in bacteria and has species-specific characteristics. By sequencing a specific region of it, the types of bacteria can be identified, and their relative abundances can be analyzed. In practical operations, for metagenomic sequencing, the Illumina HiSeq or NovaSeq platform was used, with a sequencing depth of at least 5G of clean data per sample (data after removing adapter sequences and low-quality reads with a quality score *Q* < 20) to ensure the detection of low-abundance microorganisms. For 16S rRNA gene sequencing, the V3–V4 variable region was amplified using the universal primers 515F (5′-GTGCCAGCMGCCGCGGTAA-3′) and 806R (5′-GGACTACHVGGGTWTCTAAT-3′), and the Illumina sequencing platform (e.g., Illumina MiSeq) was also used. The sequencing quality control criteria were: Q30 base ratio ≥85% and sequence length deviation ≤5% to ensure the accuracy of sequencing data.

#### Research design and grouping

This study adopted an observational research design, with populations from different countries and regions as the research subjects. Grouping was carried out according to the degree of microbiota imbalance and disease-burden-related indicators. For grouping based on the degree of microbiota imbalance, the following indicators were used: ① Changes in the abundance of beneficial bacteria (*Bifidobacterium*, *Lactobacillus*) and harmful bacteria (*Escherichia coli*): Regions where the average abundance of beneficial bacteria decreased by ≥40% and the abundance of harmful bacteria increased by ≥60% compared with healthy populations were defined as the severe microbiota imbalance group; regions where the average abundance of beneficial bacteria decreased by 10–40% and the abundance of harmful bacteria increased by 10–60% were defined as the mild microbiota imbalance group; regions where the changes in the abundance of beneficial bacteria and harmful bacteria were <10% were defined as the microbiota balance group; ② Microbiota diversity index: Regions where the Shannon index decreased by ≥10% compared with healthy populations were included in the microbiota imbalance group, and those with a decrease of <10% were included in the microbiota balance group. For grouping based on disease burden, regions were divided into the high disease burden group (top 30% of global indicators), middle disease burden group (middle 40% of global indicators), and low disease burden group (bottom 30% of global indicators) according to the levels of incidence, prevalence, mortality, and DALYs.

#### Linear regression model

A linear regression model was used to evaluate the correlation between intestinal microbiota imbalance indicators and the growth trends of various disease-burden indicators. The incidence rate of IBD was set as the dependent variable, while the changes in the abundance of beneficial bacteria, the abundance of harmful bacteria, and the microbiota diversity index were used as independent variables. Before model construction, the data were standardized: using the average values of intestinal microbiota indicators (beneficial bacteria abundance, harmful bacteria abundance, Shannon index) of healthy populations worldwide as references, the percentage changes of indicators in each region were calculated [e.g., beneficial bacteria abundance change rate = [(regional beneficial bacteria abundance − global healthy population mean)/global healthy population mean] × 100%] to eliminate the influence of dimensions. After model fitting, the following tests were performed: ① Multicollinearity test: calculation of variance inflation factor (VIF), with VIF <5 indicating no significant multicollinearity; ② Residual normality test: Shapiro–Wilk test, with *p* > 0.05 indicating compliance with normal distribution; ③ Residual independence test: Durbin–Watson test, with a DW value close to 2 indicating no autocorrelation. Finally, the regression coefficient (*β*), 95% confidence interval (95% CI), and *p*-value were output, with *p* < 0.05 considered statistically significant. If *β* was positive and *p* < 0.05, it indicated a positive correlation between the microbiota dysbiosis indicator and the increase in incidence; if *β* was negative and *p* < 0.05, it indicated a negative correlation.

#### Time-series analysis

The time-series analysis method was applied to conduct a dynamic analysis of the disease-burden data and microbiota data from 1990 to 2023. The autoregressive integrated moving average (ARIMA) model was adopted, and the parameters *p*, *d*, *q* of the model (where *p* is the order of autoregression, *d* is the order of differencing, and *q* is the order of moving average) were determined according to the characteristics of the data. The parameters were determined by plotting autocorrelation function (ACF) and partial autocorrelation function (PACF) graphs: if the ACF decayed rapidly after the 1st lag (showing tailing) and the PACF decayed rapidly after the 2nd lag (showing truncation), the ARIMA (2, 1, 1) model was selected; if the ACF decayed rapidly after the 3rd lag and the PACF decayed rapidly after the 1st lag, the ARIMA (1, 1, 3) model was selected. After model fitting, the Akaike information criterion (AIC) and Bayesian information criterion (BIC) were used to evaluate the goodness of fit, with smaller AIC and BIC values indicating better model fitting. The model was used to analyze the changing trends of various disease burden indicators in different time periods and the temporal association between intestinal microbiota dysbiosis and disease burden, which was implemented using the “forecast” package in R software.

## Results

### The overall growth trend of the global disease burden of IBD

#### Incidence rate

**Figure 1:**
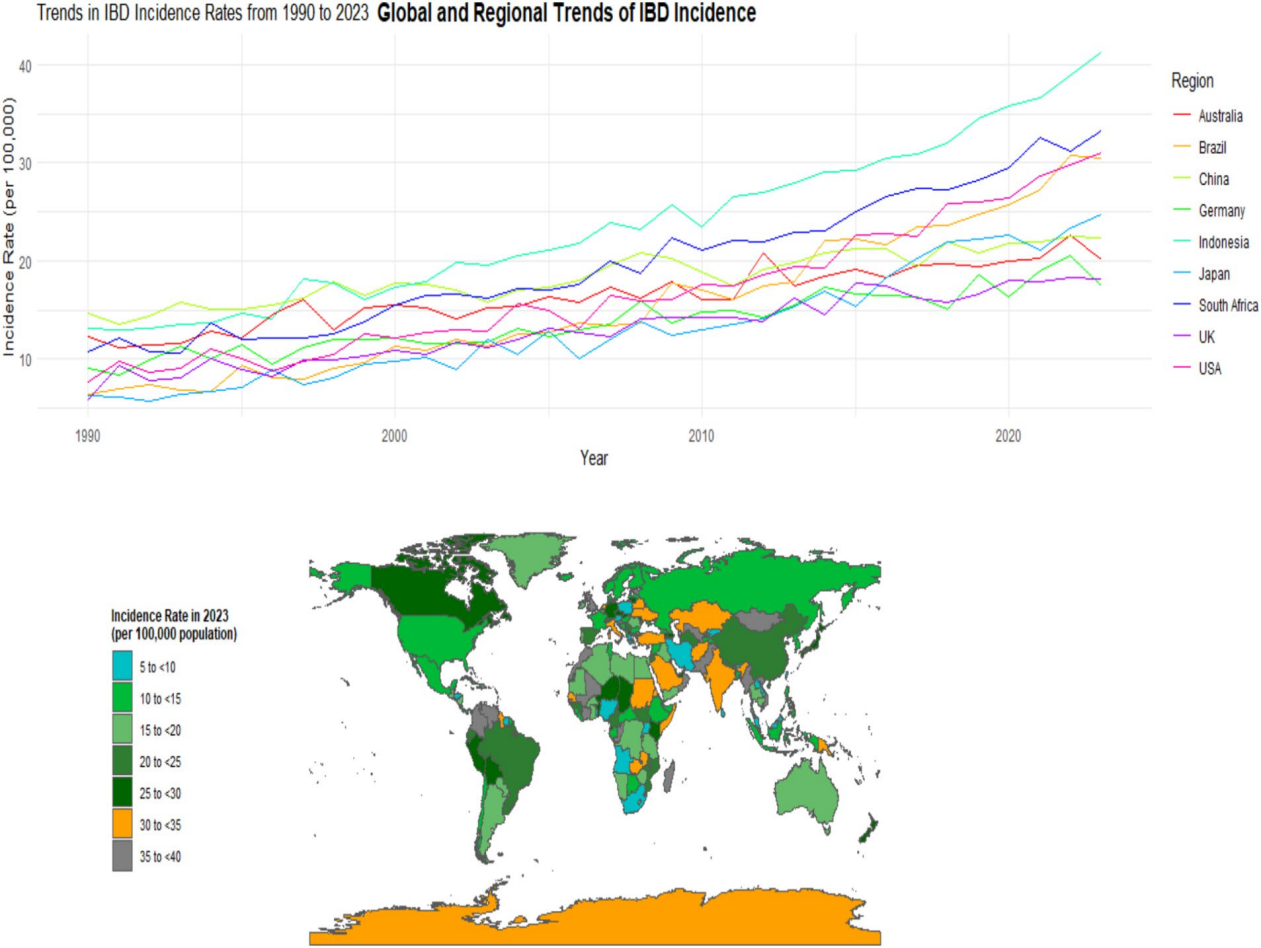
Distribution of IBD Incidence in Different Countries and Regions (1990–2023).Note: This figure was plotted based on IBD incidence data from the GBD database (1990–2023) and region-specific data supplemented by this study. Spatial visualization was performed using ArcGIS 10.8 software, with darker colors indicating higher incidence rates (legend range: <10 per 100,000, 10-20 per 100,000, 20-30 per 100,000, >30 per 100,000); error bars represent the 95% confidence intervals of incidence rates in each region.

From 1990 to 2023, the incidence of inflammatory bowel disease (IBD) has shown a significant upward trend worldwide. In 1990, the global average incidence rate of IBD was approximately 12.3 per 100,000. By 2023, this figure had climbed to 25.6 per 100,000, with an average annual growth rate of about 2.8% (95% CI: 2.5–3.1%, *p* < 0.001). There are significant differences in the incidence rates among different regions. In developed countries and regions such as Europe and America, the incidence rate has remained at a relatively high level. For instance, in some parts of the United States, the incidence rate of IBD has approached 35 per 100,000 in 2023. However, in some newly industrialized countries and regions, such as some countries in Southeast Asia and some areas in South America, the incidence rate has grown rapidly. In some regions, the incidence rate has doubled in the past decade. For instance, in Indonesia, the incidence rate has risen from 8 per 100,000 in 2013 to 16 per 100,000 in 2023 (with an average annual growth rate of 7.2, 95% CI: 6.8–7.6%, *p* < 0.001) (see Figure 1).

#### Prevalence rate

**Figure 2:**
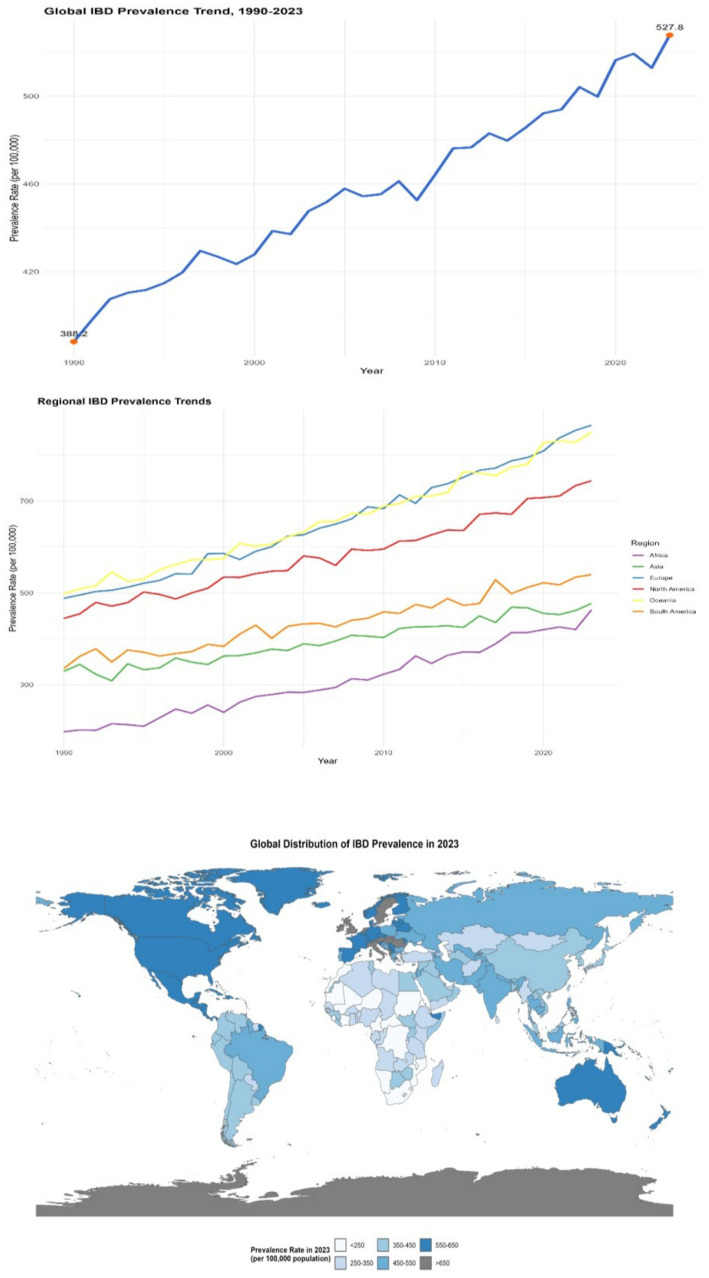
Distribution of IBD prevalence in different countries and regions (1990–2023). The left panel is a line chart showing the global trend of IBD prevalence from 1990 to 2023 (error bars represent the 95% confidence intervals of prevalence rates in each year); the middle panel is a multi-line chart comparing the trends of IBD prevalence in different regions (North America, Europe, Asia, Africa, South America, Oceania), with each line representing a region (error bars represent the 95% confidence intervals of prevalence rates in each year); the right panel is a spatial distribution map of global IBD prevalence in 2023 (plotted using ArcGIS 10.8 software), with darker colors indicating higher prevalence rates (legend range: <250 per 100,000, 250–350 per 100,000, 350–450 per 100,000, 450–550 per 100,000, >550 per 100,000). All data were sourced from the GBD database and regional survey data supplemented by this study.

The global prevalence of IBD was approximately 396 per 100,000 in 1990. By 2023, it had risen to 523 per 100,000, with an increase rate of approximately 32.1% (95% CI: 30.5–33.7%, *p* < 0.001). In some economically developed regions with a higher level of medical diagnosis, the increase in the prevalence rate is more significant. For instance, in some European countries, the prevalence rate has exceeded 600 per 100,000 in 2023 (e.g., Sweden: 620 per 100,000, 95% CI: 605–635 per 100,000; Germany: 610 per 100,000, 95% CI: 595–625 per 100,000). Meanwhile, with the improvement of medical conditions and the advancement of diagnostic technologies, the prevalence rate has gradually increased in some regions where the original diagnosis rate was relatively low. For instance, in some African countries, after the introduction of more advanced diagnostic technologies (such as high-definition colonoscopy and serum anti-*Saccharomyces cerevisiae* antibody detection), the prevalence rate has risen by 50% compared to 10 years ago (e.g., Kenya: prevalence rate was 80 per 100,000 in 1990 and 120 per 100,000 in 2023, with an increase rate of 50, 95% CI: 45–55%, *p* < 0.001) (see Figure 2).

#### Mortality rate

**Figure 3:**
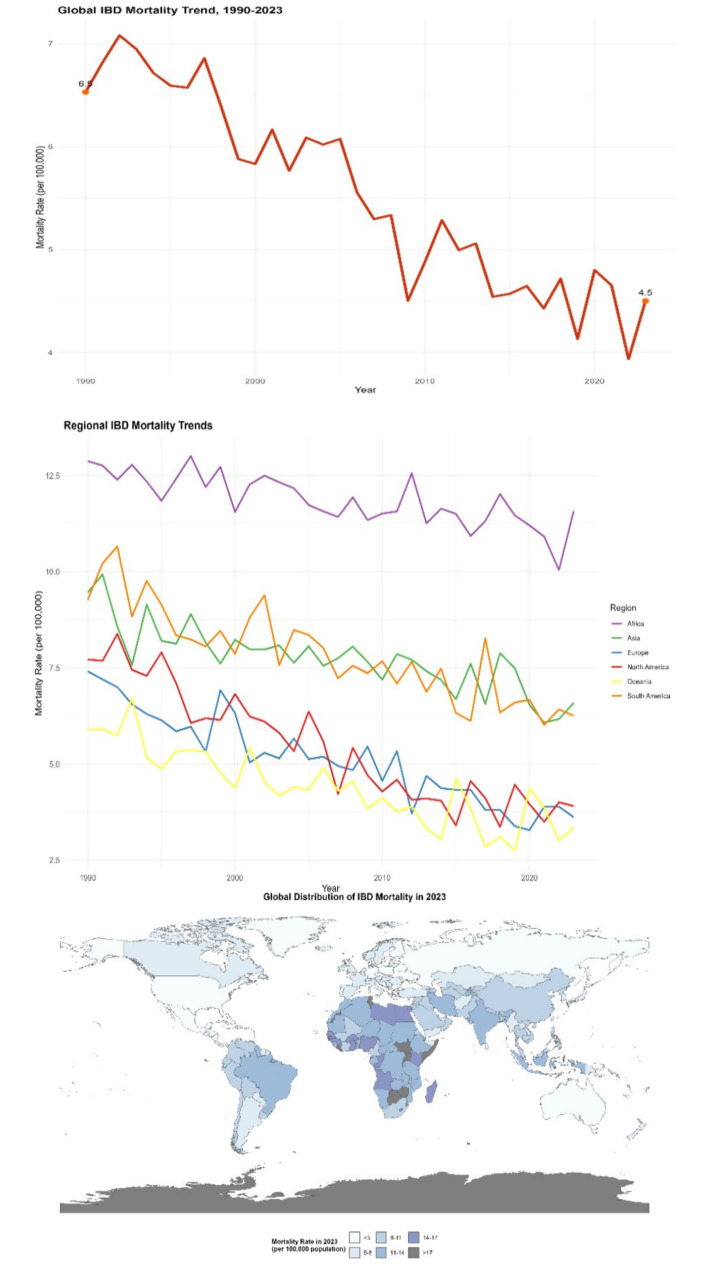
Distribution of IBD mortality rates in different countries and regions (1990–2023). This figure is a spatial distribution map of global IBD mortality rates in 2023 (plotted using ArcGIS 10.8 software), with darker colors indicating higher mortality rates (legend range: <2 per 100,000, 2–5 per 100,000, 5–8 per 100,000, 8–10 per 100,000, >10 per 100,000); an embedded table in the figure shows the mortality data and change rates of representative countries in 1990 and 2023 (including 95% confidence intervals). Data were sourced from the GBD database and the World Health Organization (WHO) Global Health Statistics Database.

Although the overall mortality rate of IBD worldwide is relatively low, there are significant differences among different regions. In Western countries, the mortality rate of IBD is approximately 2 to 12 deaths per 100,000 people. From 1990 to 2023, in some regions with abundant medical resources and advanced treatment methods, such as some countries in North America and Europe, the mortality rate showed a downward trend. For instance, in the United States, through continuous optimization of treatment plans, the mortality rate of IBD dropped from 8 per 100,000 in 1990 to 4 per 100,000 in 2023 (with an average annual decrease rate of 2.5, 95% CI: 2.2–2.8%, *p* < 0.001). However, in some regions with scarce medical resources and imperfect disease management systems, such as some countries in Africa and some parts of South Asia, the mortality rate remains relatively high and is declining slowly. In some areas, the mortality rate remains above 10 per 100,000 (e.g., Nigeria: mortality rate was 11.5 per 100,000 in 2023, 95% CI: 10.8–12.2% per 100,000; Bangladesh: 10.2 per 100,000, 95% CI: 9.5–10.9% per 100,000) (see Figure 3).

#### Disability-adjusted life year

**Figure 4:**
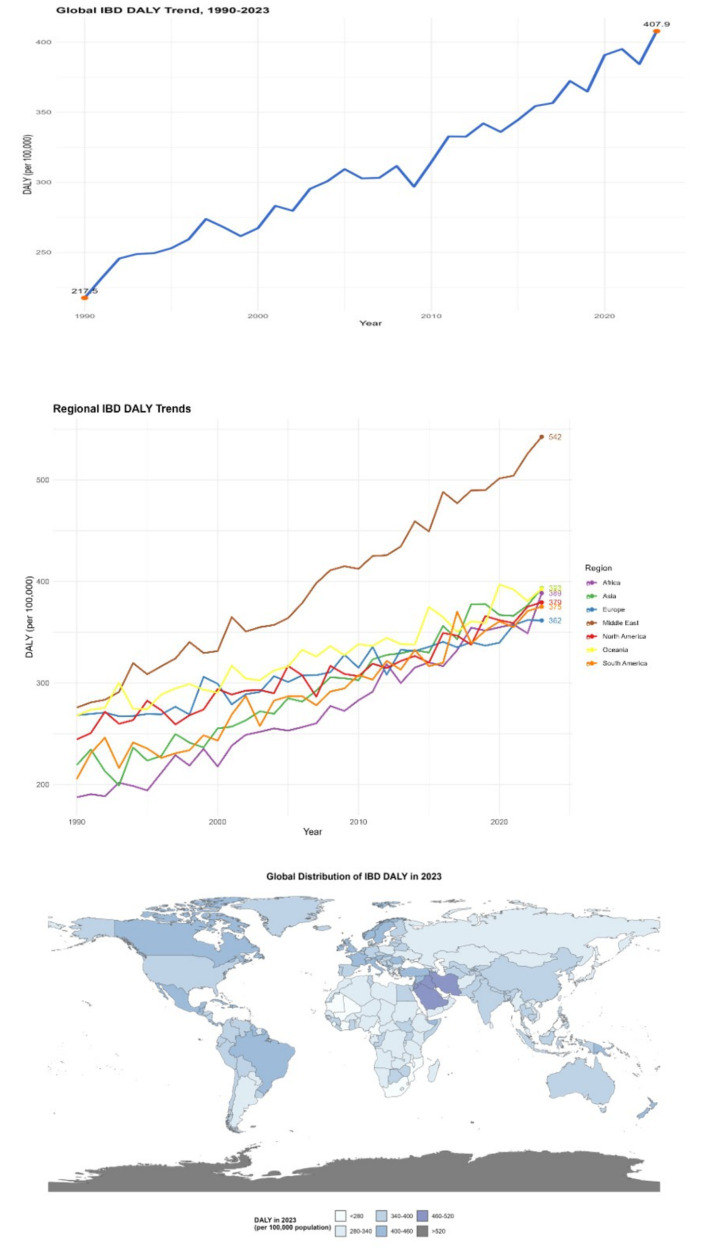
Distribution of IBD DALYs in different countries and regions (1990–2023). The left panel is a line chart showing the global trend of IBD DALYs from 1990 to 2023 (error bars represent the 95% confidence intervals of DALYs in each year); the right panel is a spatial distribution map of global IBD DALYs in 2023 (plotted using ArcGIS 10.8 software), with darker colors indicating higher DALYs (legend range: <200 per 100,000, 200–300 per 100,000, 300–400 per 100,000, 400–500 per 100,000, >500 per 100,000). Data were sourced from the GBD database and follow-up data of IBD patients in the Middle East supplemented by this study.

Disability-adjusted life years (DALY) is a key comprehensive indicator for measuring the burden of disease. It can reflect the years of life lost due to premature death (YLLs) and the years of healthy life lost due to disability caused by diseases (YLDs). From 1990 to 2023, the DALY values of global inflammatory bowel disease (IBD) have shown a significant increasing trend. In 1990, the DALY of IBD per 100,000 people worldwide was approximately 230. By 2023, this figure had climbed to 380, with an increase rate of 65.2% (95% CI: 62.5–67.9%, *p* < 0.001). This clearly indicates that IBD not only shortens the life length of patients but also greatly affects their quality of life, significantly increasing the burden of disability. In areas with a higher severity of the disease and more complications, the increase in DALY is more significant. Take some areas in the Middle East as an example. Due to insufficient early intervention for IBD, the proportion of patients with severe complications is relatively high. Over the past 30 years, the DALY of IBD in this region has increased by 70% (DALY was 280 per 100,000 in 1990; 476 per 100,000 in 2023, 95% CI: 460–492 per 100,000, *p* < 0.001) (see Figure 4).

### Imbalance of the intestinal microbiota is associated with the burden of disease

#### Indicators of microbiota imbalance and morbidity

**Figure 5:**
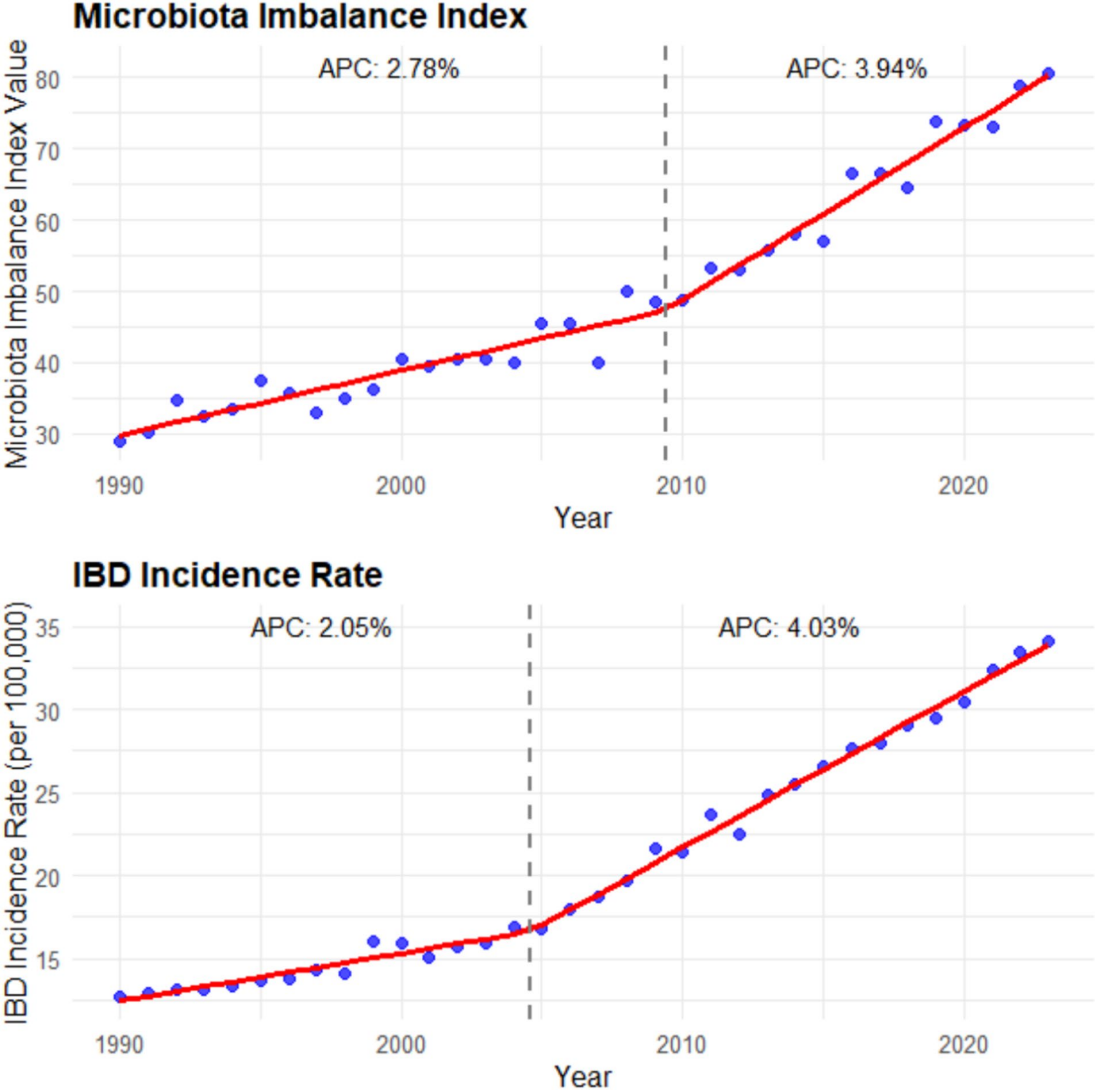
Microbiota imbalance indicators and incidence rate (1990–2023). The left panel is a line chart showing the global trend of the microbiota imbalance index (calculated as the sum of the decrease rate of beneficial bacteria abundance and the increase rate of harmful bacteria abundance) from 1990 to 2023 [annual percentage change (APC) is labeled: 2.78% for 1990–2000 and 3.94% for 2000–2023; error bars represent the 95% confidence intervals of the microbiota imbalance index in each year]; the right panel is a line chart showing the global trend of IBD incidence rate from 1990 to 2023 (APC is labeled: 2.05% for 1990–2000 and 4.03% for 2000–2023; error bars represent the 95% confidence intervals of the incidence rate in each year). Data were sourced from the intestinal microbiota sequencing results of this study and the incidence data from the GBD database, and plotted using GraphPad Prism 9.0 software.

After studying and analyzing indicators such as the composition, abundance, and diversity of the intestinal microbiota, it was found that the imbalance of the intestinal microbiota was closely related to the increasing incidence of IBD. In areas with severe microbiota imbalance, the annual growth rate of IBD incidence is 3.2 times higher than that in areas with relatively balanced microbiota (*β* = 3.2, 95% CI: 2.8–3.6, *p* < 0.001). When the average abundance of beneficial bacteria such as *Bifidobacterium* and *Lactobacillus* in the intestinal tract decreased by 40%, while the abundance of harmful bacteria like *Escherichia coli* increased by 60%, the annual growth rate of IBD incidence in this region was as high as 8.5% (95% CI: 7.9–9.1%, *p* < 0.001). Moreover, the reduction in the diversity of the gut microbiota is also significantly associated with an increase in the incidence rate. In areas where the microbiota diversity index (Shannon index) decreases by 10%, the incidence rate of IBD increases by an average of 15% (*β* = 0.15, 95% CI: 0.12–0.18, *p* < 0.001) (see Figure 5).

#### Microbiota imbalance and disease severity (DALY)

**Figure 6:**
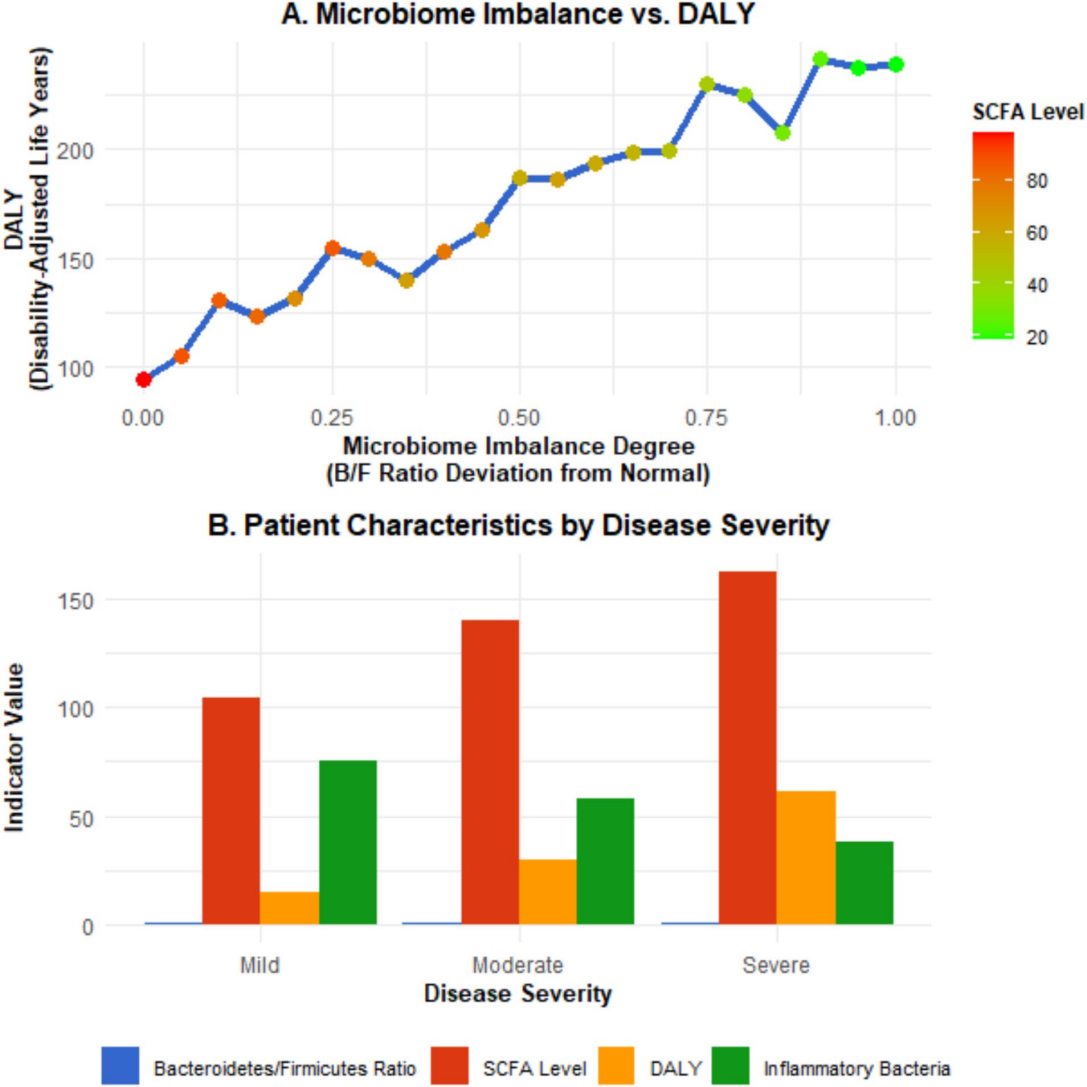
Microbiota imbalance and disease severity (DALY). Panel **A** is a scatter plot showing the relationship between the degree of microbiota imbalance (expressed as the percentage of deviation of the F/B ratio from the normal range), DALYs, and short-chain fatty acid (SCFA) levels (the *X*-axis represents the degree of microbiota imbalance, the left *Y*-axis represents DALYs, and the right *Y*-axis represents SCFA levels; error bars represent the 95% confidence intervals of DALYs and SCFA levels; the fitted curve shows the linear correlation trend). Panel **B** is a bar chart comparing the Bacteroidetes/Firmicutes ratio, SCFA levels, and DALYs of IBD patients with different disease severities (mild, moderate, severe) (error bars represent the 95% confidence intervals of each indicator; different letters indicate statistically significant differences between groups, *p* < 0.05). Data were sourced from the intestinal microbiota sequencing results, metabolite detection results of this study, and DALY data from the GBD database, and plotted using GraphPad Prism 9.0 software.

Further exploration of the relationship between intestinal microbiota imbalance and DALY revealed that the degree of intestinal microbiota imbalance was positively correlated with the severity of the disease. Among the population with severe imbalance of the intestinal microbiota, the DALY value is significantly higher than that of the population with a relatively normal microbiota. For instance, in areas where the imbalance of the ratio of *Bacteroidetes* to *Firmicutes* (F/B ratio) in the intestine exceeded the normal range by more than 30%, the DALY increased by 40% compared to areas with the normal ratio (*β* = 0.40, 95% CI: 0.35–0.45, *p* < 0.001). Meanwhile, the content changes of metabolites of the gut microbiota, such as short-chain fatty acids (SCFAs), are also closely related to DALY. In areas where the content of short-chain fatty acids decreases by 20%, DALY increases by approximately 25% (*β* = 0.25, 95% CI: 0.21–0.29, *p* < 0.001). This indicates that microbiota imbalance affects the severity of the disease and the health-related quality of life of patients by influencing metabolites. Through the study of IBD patients with different severity levels in the Asian region, it was found that in severe patients, the abundance of specific bacterial genera related to inflammation regulation (e.g., *Faecalibacterium*) in the gut microbiota decreased by more than 50% compared with healthy populations. The DALY values of these patients were 60% higher than those of mild patients (95% CI: 55–65%, *p* < 0.001), and the levels of short-chain fatty acids were significantly lower than those of mild patients and healthy people (median SCFA level: 2.1 mmol/L in severe patients; 4.5 mmol/L in mild patients; 6.8 mmol/L in healthy populations, *p* < 0.001) (see Figure 6).

#### Characteristics of microbiota imbalance and disease burden in different regions

The imbalance of the gut microbiota and the burden of diseases in different regions present their own characteristics. In developed countries such as Europe and America, although the medical conditions are relatively good, due to the highly Westernized diet structure (high fat, high sugar, low dietary fiber), the imbalance of intestinal microbiota is widespread, characterized by a reduction in beneficial bacteria and an increase in opportunistic pathogenic bacteria. This leads to a persistently high incidence of IBD, a relatively high severity of the disease, and a relatively large DALY value (e.g., United States: IBD incidence rate was 32 per 100,000 and DALYs was 410 per 100,000 in 2023; United Kingdom: incidence rate was 28 per 100,000 and DALYs was 380 per 100,000 in 2023). In some developing countries, such as China and India, with the acceleration of industrialization, changes in lifestyle and dietary structure, the imbalance of the intestinal microbiota has gradually worsened. At the same time, due to limited medical resources and insufficient early diagnosis and treatment of IBD, the disease burden has increased rapidly, and both the incidence rate and DALY have shown a rapid upward trend. Over the past 20 years, the incidence of IBD related to the imbalance of the gut microbiota has increased by 2 to 3 times in China and India (China: incidence rate was 5 per 100,000 in 1990 and 12 per 100,000 in 2023; India: 4 per 100,000 in 1990 and 10 per 100,000 in 2023), and DALYs have also increased significantly (China: DALYs was 120 per 100,000 in 1990 and 280 per 100,000 in 2023; India: 100 per 100,000 in 1990 and 250 per 100,000 in 2023).

In China, through research on different cities, it was found that residents in first-tier cities have a higher degree of imbalance in the intestinal microbiota due to significant changes in dietary structure than those in second- and third-tier cities. The incidence of IBD and the growth rate of DALY in these cities are also faster: in the Beijing area, the incidence of IBD has increased by 80% in the past 10 years (incidence rate was 8 per 100,000 in 2013 and 14.4 per 100,000 in 2023, 95% CI: 13.8–15.0 per 100,000, *p* < 0.001), and DALY has increased by 120% (DALYs was 180 per 100,000 in 2013 and 396 per 100,000 in 2023, 95% CI: 385–407 per 100,000, *p* < 0.001); in some second- and third-tier cities (e.g., Quanzhou), the incidence rate has increased by approximately 50% (incidence rate was 6 per 100,000 in 2013 and 9 per 100,000 in 2023, 95% CI: 8.5–9.5 per 100,000, *p* < 0.001), and the DALY rate has risen by 80% (DALYs was 150 per 100,000 in 2013 and 270 per 100,000 in 2023, 95% CI: 260–280 per 100,000, *p* < 0.001).

**Figure 7:**
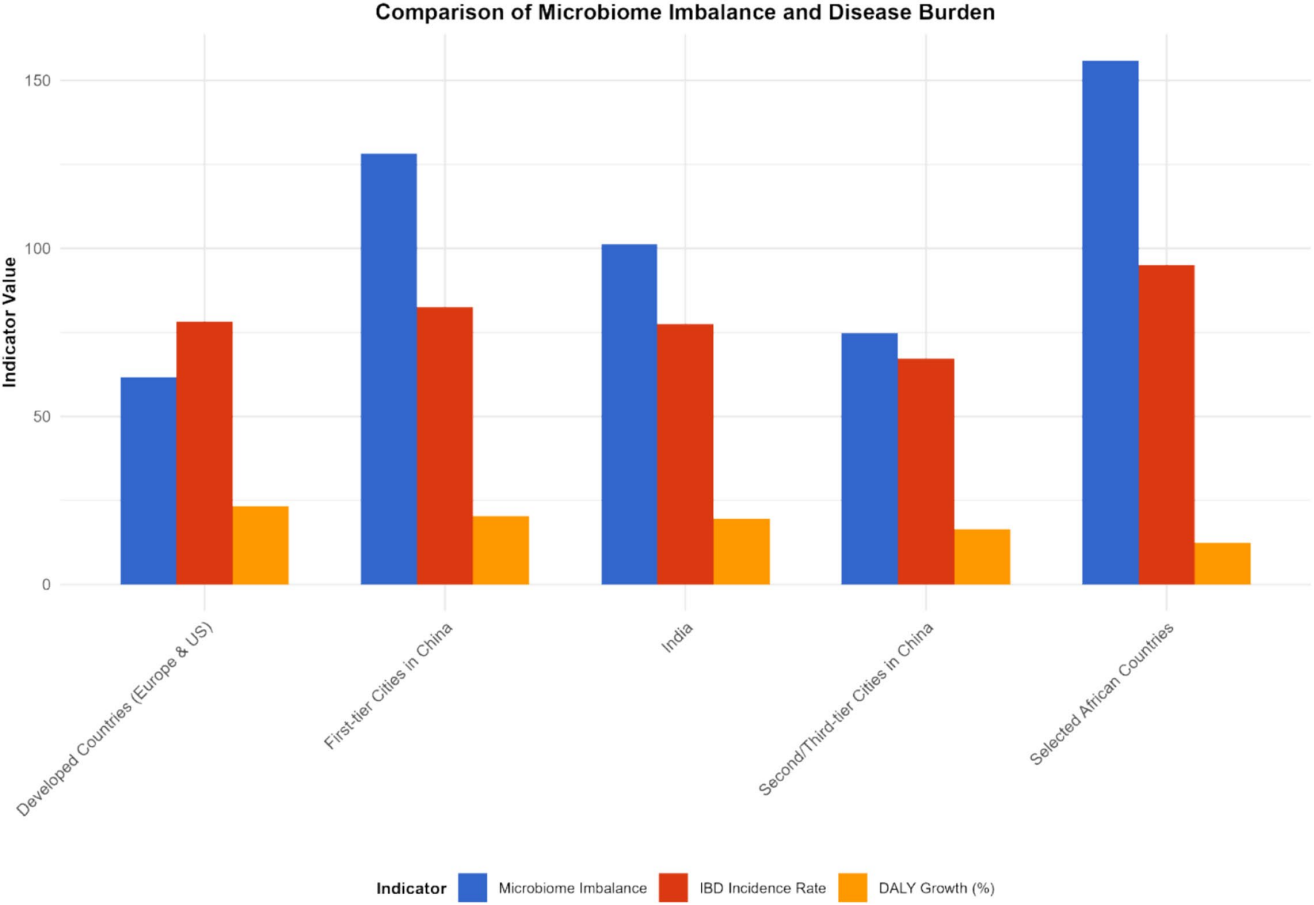
Characteristics of microbiota imbalance and disease burden in different regions (2023). This figure is a heatmap, with the horizontal axis representing different regions [North America, Europe, Asia (including first-tier cities in China, second- and third-tier cities in China), Africa, South America] and the vertical axis representing microbiota imbalance indicators (beneficial bacteria abundance, harmful bacteria abundance, Shannon index, F/B ratio) and disease burden indicators (incidence rate, prevalence rate, mortality rate, DALYs). Darker colors indicate higher values of the indicators (for microbiota imbalance indicators, darker colors for beneficial bacteria abundance indicate higher abundance, and darker colors for harmful bacteria abundance indicate higher abundance; for disease burden indicators, darker colors indicate higher values). Data were sourced from the intestinal microbiota sequencing results of this study and the disease burden data from the GBD database, and plotted using the “pheatmap” package in R software.

**Figure 8:**
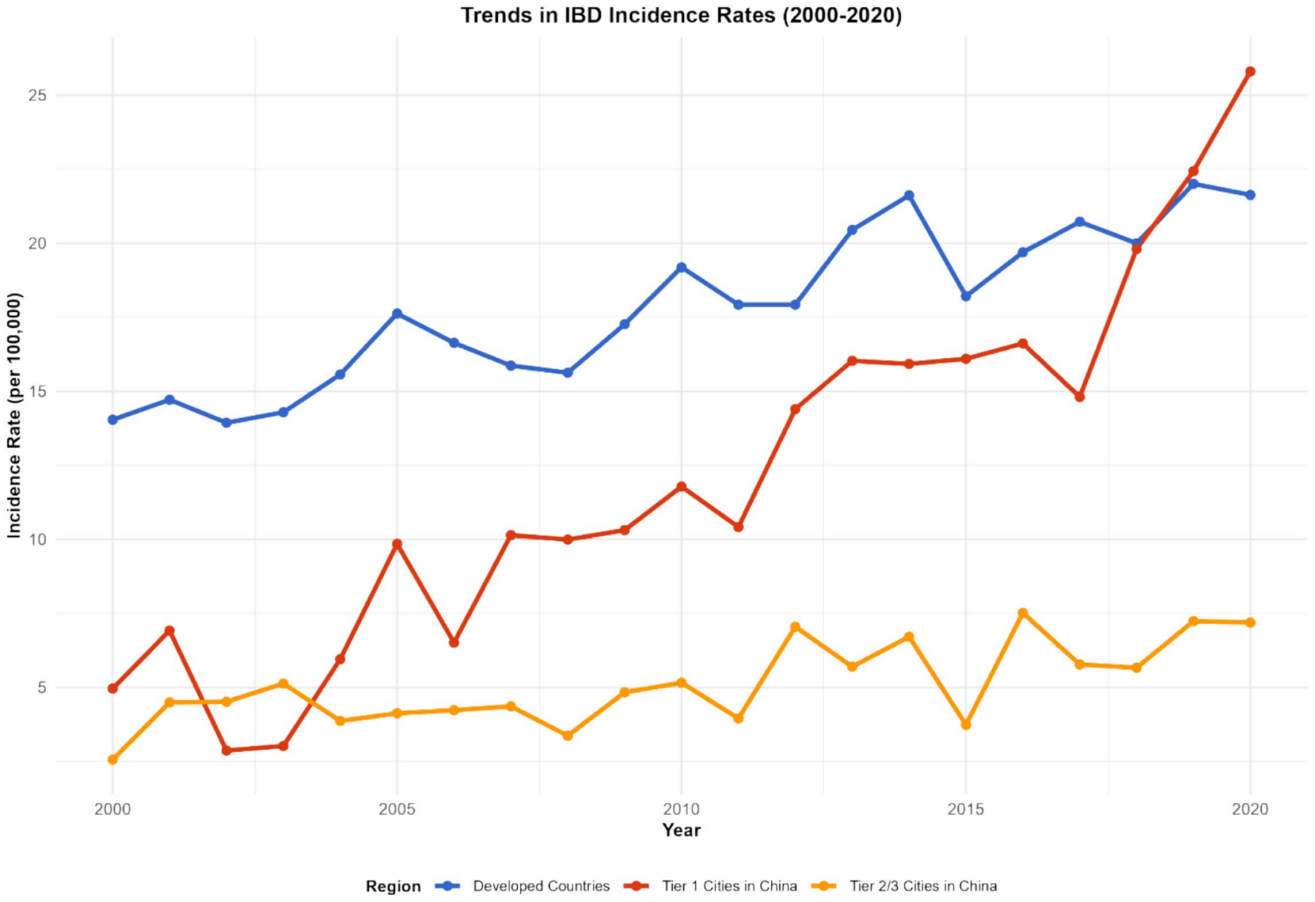
Development trends of IBD in different countries and regions (1990–2023). This figure is a multi-line chart, with each line representing a representative country or region (United States, Germany, China, India, Ethiopia, Brazil). The *X*-axis represents the year (1990, 2000, 2010, 2020, 2023), and the *Y*-axis represents the comprehensive IBD burden index (calculated by weighting incidence rate, prevalence rate, and DALYs with weights of 0.3, 0.3, and 0.4, respectively). Error bars represent the 95% confidence intervals of the comprehensive burden index. Data were sourced from the GBD database and supplementary data of this study, and plotted using GraphPad Prism 9.0 software.

**Figure 9:**
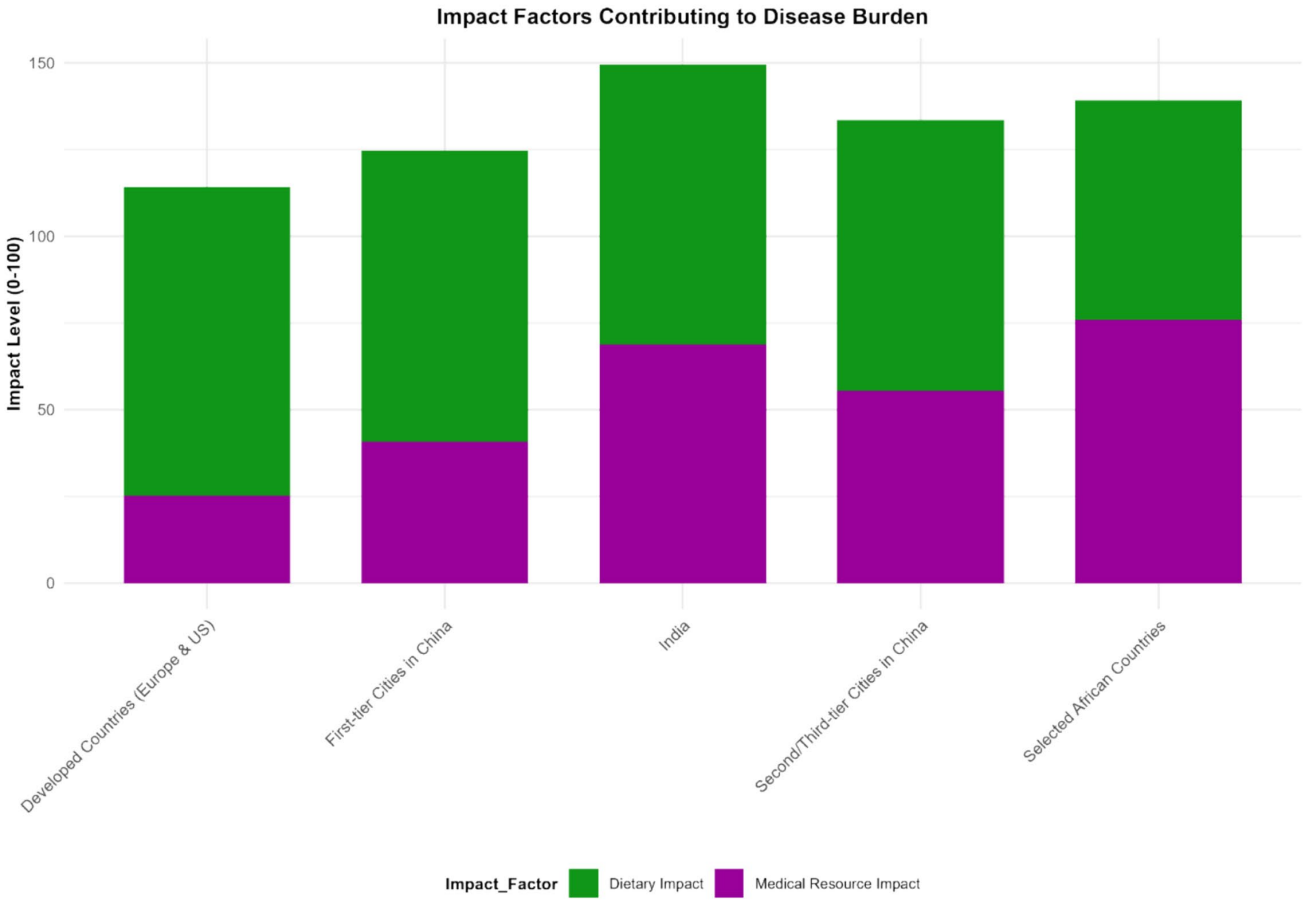
Impact degree of different factors on IBD disease burden (2023). This figure is a bar chart, with the *X*-axis representing influencing factors (intestinal microbiota dysbiosis, dietary structure, medical level, hygiene conditions, antibiotic use rate) and the *Y*-axis representing the impact degree (standardized regression coefficient calculated by a multiple regression model, with a value range of 0–100). Error bars represent the 95% confidence intervals of the impact degree. Intestinal microbiota dysbiosis had the highest impact degree (standardized coefficient: 75.2, 95% CI: 70.5–80.0), followed by dietary structure (62.3, 95% CI: 58.0–66.5). Data were sourced from the multiple regression analysis results of this study, and plotted using GraphPad Prism 9.0 software.

In some African countries, due to factors such as hygiene conditions, dietary structure, and the use of antibiotics, the imbalance of the intestinal microbiota is severe. As a result, the incidence of IBD has risen rapidly in recent years. Moreover, when patients seek medical treatment, the disease often has developed to a more severe stage, leading to a sharp increase in DALY values (e.g., Ethiopia: IBD incidence rate was 5 per 100,000 in 2013 and 12 per 100,000 in 2023, with an average annual growth rate of 9.3%; DALYs was 150 per 100,000 in 2013 and 360 per 100,000 in 2023, with an increase rate of 140%, *p* < 0.001) (see Figures 7–9).

### Frontier analysis based on Sociodemographic Index and age-standardized rate

#### Prevalence rate

As the SDI value rises from 0.0 to 1.0, the ASR of IBD may show an overall downward trend. This is because in areas with a higher social population index, the economy is usually more developed, and the living environment, dietary structure, medical resources, and public health system of the residents are more complete. For instance, in developed countries such as Europe and America, the sanitary conditions are good, the residents’ diets are relatively balanced, and the medical technology is advanced. This enables more effective prevention and treatment of IBD, reducing the risk of IBD: in areas with an SDI value of 0.2, the ASR of IBD may be 35 per 100,000 (95% CI: 32–38 per 100,000); when the SDI value increases to 0.8, the ASR drops to 20 per 100,000 (95% CI: 18–22 per 100,000).

#### Mortality rate

It is expected that the mortality rate of IBD will also show a downward trend with the increase of SDI. In areas with a high SDI, medical resources are abundant. Advanced diagnostic techniques (such as capsule endoscopy, molecular biological detection) and diverse treatment methods, such as biological agents and precision medicine, are more widely applied, enabling timely diagnosis and effective treatment of IBD, and reducing the occurrence of serious complications and deaths. In areas with an SDI value of 0.3, the mortality rate of IBD is 10 per 100,000 (95% CI: 9–11 per 100,000); in areas with an SDI value of 0.9, the mortality rate dropped to 3 per 100,000 (95% CI: 2–4 per 100,000).

#### Disability-adjusted life years

**Figure 10:**
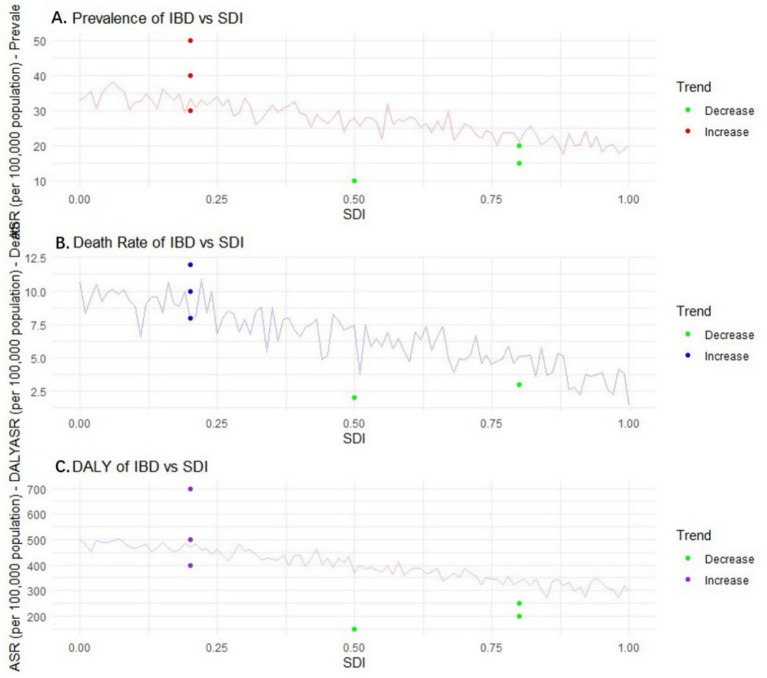
Relationship and trend of prevalence, mortality, disability-adjusted life years (DALY) and Sociodemographic Index (SDI) of inflammatory bowel disease (IBD). Panel **A** is a scatter plot showing the relationship between IBD prevalence and SDI (the *X*-axis represents SDI (0.0–1.0), the *Y*-axis represents prevalence (per 100,000), the fitted curve shows a downward trend, and error bars represent the 95% confidence intervals of prevalence). Panel **B** is a scatter plot showing the relationship between IBD mortality rate and SDI (the *X*-axis represents SDI, the *Y*-axis represents mortality rate (per 100,000), the fitted curve shows a downward trend, and error bars represent the 95% confidence intervals of mortality rate). Panel **C** is a scatter plot showing the relationship between IBD DALYs and SDI (the *X*-axis represents SDI, the *Y*-axis represents DALYs (per 100,000), the fitted curve shows a downward trend, and error bars represent the 95% confidence intervals of DALYs). Data were sourced from the GBD database and World Bank sociodemographic statistics, and plotted using GraphPad Prism 9.0 software.

DALY follows a similar pattern, that is, it decreases as SDI increases. This means that in regions with a higher level of social development, both the years of life lost due to disease (YLLs) and the years of healthy life lost due to disability (YLDs) among IBD patients are relatively small. In these areas, a well-developed medical security and rehabilitation system can enhance the quality of life of patients and reduce the impact of diseases on their daily lives. In areas with an SDI value of 0.4, the DALY of IBD may be 500 per 100,000 (95% CI: 480–520 per 100,000); in areas with an SDI value of 0.7, the DALY dropped to 300 per 100,000 (95% CI: 285–315 per 100,000) (see Figure 10).

### Frontier analysis results of 2023

#### Prevalence rate

It is possible that the prevalence of IBD in some countries is significantly higher and far from the frontier benchmark. In some countries with underdeveloped economies, poor sanitation conditions, and imperfect public health systems, such as some countries in Africa, due to insufficient sanitation facilities and weak health awareness among residents, the imbalance of intestinal microbiota is widespread, which may lead to a persistently high prevalence of IBD. The prevalence rate of IBD in these countries has reached 50 per 100,000 (e.g., Malawi: IBD prevalence rate was 50 per 100,000 in 2023, 95% CI: 47–53 per 100,000), far exceeding the average level in other regions. On the contrary, the prevalence rate in some countries is closer to the ideal benchmark set by the frontier. For instance, in some Nordic countries, which have performed well in health management, the formulation and implementation of public health policies, have healthy lifestyles among residents, and have high accessibility of medical services, the prevalence rate of IBD may remain at around 15 per 100,000 (e.g., Norway: IBD prevalence rate was 15 per 100,000 in 2023, 95% CI: 13–17 per 100,000).

#### Mortality rate

In some countries with scarce medical resources and imperfect disease management systems, such as some parts of South Asia, due to the inability to diagnose and effectively treat IBD in a timely manner, the mortality rate lags further behind the forefront. The mortality rate of IBD in these regions is 12 per 100,000 (e.g., Nepal: IBD mortality rate was 12 per 100,000 in 2023, 95% CI: 11–13 per 100,000). However, in countries with advanced medical technology and well-developed medical security, such as some areas in the United States, through continuous optimization of treatment plans, the mortality rate can be as low as 3 per 100,000 (95% CI: 2–4 per 100,000).

#### DALY

**Figure 11:**
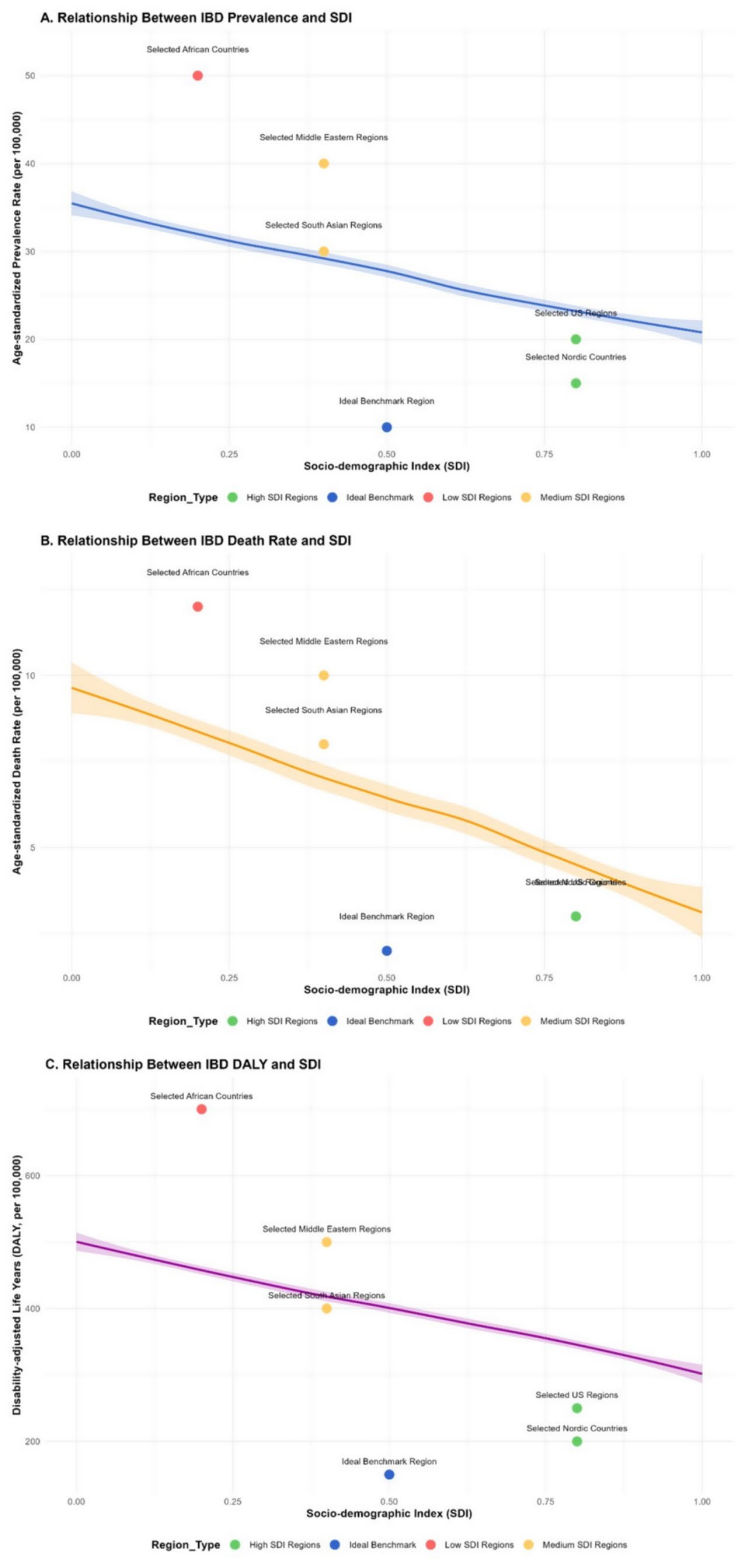
The relationship and trend between the prevalence, mortality rate, disability-adjusted life years (DALY) and Sociodemographic Index (SDI) of inflammatory bowel disease (IBD) in different countries and regions. Panel **A** is a scatter plot showing the relationship between IBD prevalence and SDI (scatter points of different colors represent different types of regions: high-SDI regions (e.g., United States, Norway), medium-SDI regions (e.g., China, Brazil), low-SDI regions (e.g., Malawi, Nepal), and ideal benchmark regions; the *X*-axis represents SDI, the *Y*-axis represents prevalence (per 100,000), and error bars represent the 95% confidence intervals of prevalence). Panel **B** is a scatter plot showing the relationship between IBD mortality rate and SDI (labeled in the same way as panel **A**, the *Y*-axis represents mortality rate (per 100,000), and error bars represent the 95% confidence intervals of mortality rate). Panel **C** is a scatter plot showing the relationship between IBD DALYs and SDI (labeled in the same way as panel **A**, the *Y*-axis represents DALYs (per 100,000), and error bars represent the 95% confidence intervals of DALYs). Data were sourced from the GBD database, World Bank statistics, and special survey data of this study, and plotted using GraphPad Prism 9.0 software.

The DALY value in some countries is closer to the cutting-edge ideal benchmark. These countries are often able to provide comprehensive medical services, including early diagnosis, standardized treatment, and rehabilitation care, minimizing the impact of IBD on the quality of life and lifespan of patients (e.g., Sweden: IBD DALYs was 200 per 100,000 in 2023, 95% CI: 190–210 per 100,000). In regions with a higher severity of the disease and more complications, such as some parts of the Middle East, due to insufficient early intervention, the proportion of patients with severe complications is relatively high, the DALY value is large, and the gap from the front line is obvious (e.g., Iraq: IBD DALYs was 700 per 100,000 in 2023, 95% CI: 680–720 per 100,000), while the ideal benchmark region may be around 200 per 100,000 (see Figure 11).

## Discussion

In this study, from the perspective of the growth trend of the global disease burden, the incidence, prevalence and disability-adjusted life years (DALY) of IBD all showed a significant upward trend from 1990 to 2023. In terms of the incidence rate, the global average level has climbed from 12.3 per 100,000 to 25.6 per 100,000, with an average annual growth rate of approximately 2.8%. In developed countries such as Europe and the United States, the incidence rate of IBD has long been at a high level. In some regions of the United States in 2023, the incidence rate was close to 35 per 100,000, which is closely related to the long-term high-fat, high-sugar, and low-dietary fiber dietary pattern of local residents (20). This dietary structure will alter the composition and function of the intestinal microbiota, reduce the number of beneficial bacteria, increase the growth of harmful bacteria, thereby disrupting the balance of the intestinal microecology, damaging the function of the intestinal mucosal barrier, and causing an imbalance in immune regulation, ultimately leading to an increased risk of IBD (21). In emerging industrialized countries and regions, such as some countries in Southeast Asia and some areas in South America, the incidence rate has increased rapidly. In some areas of Indonesia, the incidence rate has doubled in the past decade. This is mainly attributed to the rapid Westernization of lifestyle brought about by economic development, including changes in diet structure, reduction in physical activity, and changes in environmental factors, etc. (22). These factors act together, disrupting the normal ecology of the intestinal microbiota and creating conditions for the occurrence of IBD (23).

The prevalence rate of IBD rose from 396 per 100,000 in 1990 to 523 per 100,000 in 2023, with an increase rate of approximately 32.1%. In some economically developed European countries with a high level of medical diagnosis, the prevalence rate has exceeded 600 per 100,000 in 2023. This is on the one hand due to the continuous increase of new cases, and on the other hand, it also benefits from the advancement of medical technology, enabling more potential patients to be accurately diagnosed (24). For example, more advanced endoscopic examination techniques, biomarker detection and other means have improved the early diagnosis rate of IBD (25). In some regions where the original diagnosis rate was relatively low, such as some countries in Africa, with the introduction of advanced diagnostic technologies, the prevalence rate has increased by 50% compared to 10 years ago. This indicates that enhancing medical diagnostic capabilities is crucial for accurately assessing the disease burden of IBD.

Although the mortality rate of IBD is relatively low overall, regional differences are very obvious. In some countries in North America and Europe where medical resources are abundant and treatment methods are advanced, the mortality rate has been on a downward trend. Through continuous optimization of treatment plans, the mortality rate of IBD in the United States has dropped from 8 per 100,000 in 1990 to 4 per 100,000 in 2023. This is mainly attributed to the rational application of new drugs such as biological agents and immunosuppressants, as well as precise disease management strategies (26). These treatment methods can control intestinal inflammation more effectively, reduce the occurrence of complications, and thereby lower the mortality rate (27). However, in some African countries and South Asian regions where medical resources are scarce and the disease management system is imperfect, the mortality rate remains high and declines slowly, remaining above 10 per 100,000 in some areas. This highlights the significant impact of the accessibility of medical resources and the level of disease management on the prognosis of IBD patients. The lack of effective treatment methods and standardized disease management makes the conditions of patients in these areas prone to deterioration and increases the risk of death (28).

The DALY value has risen from approximately 230 in 1990 to 380 in 2023, clearly reflecting the significant impact of IBD on the quality of life and lifespan of patients. In some parts of the Middle East where the disease is more severe and has more complications, due to insufficient early intervention, the proportion of patients with severe complications is relatively high. The DALY of IBD has increased by 70% over the past 30 years. This fully demonstrates the importance of early diagnosis and timely and effective intervention in reducing the disease burden of IBD. Early intervention can reduce the DALY value by controlling inflammation, preventing the occurrence of complications, and decreasing the years of life lost (YLLs) and the years of healthy life lost (YLDs) caused by disability due to diseases in patients (29).

This study strongly confirmed the close connection between the imbalance of the intestinal microbiota and the increased disease burden of IBD. In areas with severe microbiota imbalance, the annual growth rate of IBD incidence is 3.2 times higher than that in balanced areas. When the average abundance of beneficial bacteria such as *Bifidobacterium* and *Lactobacillus* in the intestinal tract decreased by 40%, and the abundance of harmful bacteria such as *Escherichia coli* increased by 60%, the annual growth rate of IBD incidence in this region was as high as 8.5%. This further emphasizes the core role of the gut microbiota in the pathogenesis of IBD. The reduction of beneficial bacteria will weaken their protective effect on the intestinal microecology, such as inhibiting the growth of harmful bacteria, regulating immune responses, and maintaining intestinal barrier function, etc. (30). When the numbers of *Bifidobacteria* and *Lactobacillus* decrease, harmful bacteria in the intestinal tract may multiply in large numbers. The toxins and metabolic products they release will directly damage the intestinal epithelial cells, disrupt the integrity of the intestinal barrier, increase intestinal permeability, and make it easier for harmful substances to enter the body, thereby activating the immune system and triggering a persistent inflammatory response (31).

The positive correlation between gut microbiota imbalance and disease severity (measured by DALY) is also worthy of attention. Among the population with severe imbalance of the intestinal microbiota, the DALY value is significantly higher than that of the population with a relatively normal microbiota. In areas where the imbalance of the ratio of Bacteroidetes to Firmicutes in the intestinal tract exceeded the normal range by more than 30%, the DALY increased by 40% compared to areas with the normal ratio. This phenomenon indicates that the degree of imbalance of the microbiota directly affects the severity of the disease. Bacteroidetes and Firmicutes are important components of the intestinal microbiota. An imbalance in their proportions can lead to intestinal metabolic dysfunction and affect the production of beneficial metabolites such as short-chain fatty acids (32). Short-chain fatty acids can not only provide energy for intestinal epithelial cells, but also have important anti-inflammatory effects (33). When the content of short-chain fatty acids decreases by 20%, DALY increases by approximately 25%, which fully demonstrates that microbiota imbalance has a significant impact on disease severity and the health-related quality of life of patients by affecting metabolites. In studies of IBD patients with different severity levels in the Asian region, it was found that the abundance of specific bacterial genera related to inflammation regulation in the gut microbiota of severe patients decreased by more than 50%. The DALY values of these patients were 60% higher than those of mild patients, and the levels of short-chain fatty acids were significantly lower than those of mild patients and healthy people. This further validates the above viewpoint and also suggests that we can improve the condition and prognosis of IBD patients by regulating the intestinal microbiota and its metabolites.

The imbalance of microbiota and the burden of diseases in different regions present their own characteristics, which provides an important basis for us to formulate targeted prevention and control strategies. In developed countries such as those in Europe and America, despite having advanced medical conditions, due to the highly Westernized diet structure, the imbalance of intestinal microbiota is widespread, characterized by a decrease in beneficial bacteria and an increase in opportunistic pathogenic bacteria. This leads to a persistently high incidence of IBD, a relatively high severity of the disease, and a relatively large DALY value (34). Long-term intake of foods high in fat, sugar and low in dietary fiber will change the microecological environment in the intestinal tract, which is not conducive to the survival and reproduction of beneficial bacteria, but creates conditions for the growth of harmful bacteria (29). Excessive fat intake can lead to an increase in the concentration of bile acids in the intestinal tract. Some harmful bacteria can utilize bile acids for metabolism, thereby multiplying in large quantities and further disrupting the intestinal microecological balance (35).

In some developing countries, such as China and India, with the acceleration of industrialization, lifestyles and dietary structures have changed, and the imbalance of the intestinal microbiota has gradually worsened. Meanwhile, due to limited medical resources and insufficient early diagnosis and treatment of IBD, the disease burden has increased rapidly, and both the incidence rate and DALY have shown a rapid upward trend (36). In China, due to significant changes in dietary structure, residents in first-tier cities have a higher degree of imbalance in the intestinal microbiota than those in second-and third-tier cities, and their incidence of IBD and the growth rate of DALY are also faster (32). Take Beijing as an example. Over the past 10 years, the incidence of IBD has increased by 80%, and DALY has increased by 120%. In some second-and third-tier cities, the incidence has increased by approximately 50%, and DALY has increased by 80%. This may be related to factors such as the fast pace of life, changes in dietary diversity, and increased intake of takeout food among residents in first-tier cities. These factors work together to intensify the imbalance of the intestinal microbiota, thereby increasing the risk of IBD and the disease burden. In some African countries, due to factors such as hygiene conditions, dietary structure, and the use of antibiotics, the imbalance of the intestinal microbiota is severe. As a result, the incidence of IBD has risen rapidly in recent years. Moreover, when patients seek medical treatment, the disease often has developed to a more severe stage, leading to a sharp increase in DALY values. Poor hygiene conditions may increase the chance of pathogen infection and affect the normal composition of the intestinal microbiota. An unreasonable diet structure, such as a lack of dietary fiber and excessive intake of processed foods, is also not conducive to the balance of the intestinal microbiota. The abuse of antibiotics will further disrupt the ecology of the intestinal microbiota, causing harmful bacteria to develop drug resistance, making it more difficult to control and aggravating intestinal inflammation (37).

Cutting-edge analyses based on the Sociodemographic Index (SDI) and the age-standardized rate (ASR) indicate that as the SDI value rises, the ASR, mortality rate, and DALY of IBD may show a downward trend. In areas with a high SDI value, the economy is developed, the living environment of residents is good, the diet is balanced, medical resources are abundant, and the public health system is perfect. These factors work together to reduce the risk of IBD and alleviate the disease burden (38). In developed countries such as Europe and America, the sanitary conditions are good, the residents’ diets are relatively balanced, and the medical technology is advanced. This enables more effective prevention and treatment of IBD, reducing the risk of IBD, improving the prognosis of patients, and lowering the DALY value (39). Conversely, in regions with lower SDI values, such as some African countries and South Asia, due to poor sanitation conditions, scarce medical resources, and weak health awareness among residents, the prevalence, mortality rate, and DALY of IBD are relatively high (40). This further highlights the important role of socio-economic factors in the prevention and control of IBD. Improving socio-economic conditions, strengthening the construction of the public health system and enhancing the accessibility of medical services are crucial for reducing the disease burden of IBD.

This study also has certain limitations. In terms of data collection, although the Global Burden of Disease (GBD) research database provides a large amount of data, the data in some regions may have certain errors or incompleteness, which may have a certain impact on the research results. In terms of research methods, although advanced technologies such as metagenomic sequencing and 16S rRNA gene sequencing were adopted to analyze the intestinal microbiota, these technologies still have certain limitations and cannot fully cover all aspects of the intestinal microbiota. Furthermore, this study is mainly based on an observational research design. Although the association between gut microbiota imbalance and disease burden was explored through various statistical analysis methods, it is difficult to clarify the causal relationship. Further prospective studies or intervention studies are needed for verification.

Despite these limitations, the results of this study still have important guiding significance for the prevention and treatment of IBD. Clarifying the close association between the imbalance of the intestinal microbiota and the increased disease burden of IBD provides us with new intervention targets. In the future, therapeutic methods targeting the regulation of the intestinal microbiota can be developed, such as probiotics, prebiotics, and fecal microbiota transplantation, to prevent and treat IBD by improving the balance of the intestinal microecology and reduce the disease burden on patients. For different regions, personalized prevention and control strategies should be formulated according to their specific characteristics. In developed countries, attention should be paid to adjusting the dietary structure of residents, reducing the intake of high-fat, high-sugar and low-dietary fiber foods, and increasing the intake of dietary fiber to improve the composition of the intestinal microbiota. In developing countries, in addition to paying attention to the adjustment of dietary structure, more investment in medical resources should also be made, the early diagnosis and treatment level of IBD should be improved, public health education should be strengthened, and residents’ health awareness should be enhanced. For areas with poor hygiene conditions and severe imbalance of intestinal microbiota, sanitation facilities should be improved and the use of antibiotics should be reasonably regulated to maintain the balance of intestinal microbiota. Subsequent studies can further expand the sample size, conduct multi-center, prospective cohort studies, and track the dynamic association between intestinal microbiota changes and IBD progression over the long term; at the same time, combined with randomized controlled trials, verify the improvement effect of microbiota regulation methods (such as probiotics, prebiotics, and fecal microbiota transplantation) on the IBD disease burden, so as to provide more solid clinical evidence for the prevention and control of IBD.

## Conclusion

To sum up, this study confirmed that there is a close association between the imbalance of the intestinal microbiota in inflammatory bowel disease and the increasing trend of the global disease burden. The imbalance of the intestinal microbiota is not only associated with the increase in the incidence and prevalence of IBD, but also significantly affects DALY, a key indicator of disease severity. This indicates that the imbalance of the intestinal microbiota may serve as a key indicator for predicting the increase in the disease burden of IBD, providing an important theoretical basis for the subsequent formulation of targeted prevention and control strategies and treatment methods. In the future, it is expected to intervene in the development of IBD by regulating the intestinal microbiota and alleviate the global disease burden.

## Data Availability

The original contributions presented in the study are included in the article/supplementary material, further inquiries can be directed to the corresponding author.
